# Age-Dependent Demethylation of* Sod2* Promoter in the Mouse Femoral Artery

**DOI:** 10.1155/2016/8627384

**Published:** 2016-02-16

**Authors:** Albert Nguyen, François Leblond, Maya Mamarbachi, Steve Geoffroy, Eric Thorin

**Affiliations:** ^1^Department of Pharmacology, Faculty of Medicine, Université de Montréal, Montreal, QC, Canada H3T 1J4; ^2^Research Centre, Montreal Heart Institute, 5000 Belanger Street, Montreal, QC, Canada H1T 1C8; ^3^Novartis Pharmaceuticals Canada Inc., Dorval, QC, Canada H9S 1A9; ^4^Department of Surgery, Faculty of Medicine, Université de Montréal, Montreal, QC, Canada H3T 1J4

## Abstract

We studied the age-dependent regulation of the expression of the antioxidant enzyme manganese superoxide dismutase (MnSOD encoded by* Sod2*) through promoter methylation. C57Bl/6 mice were either (i) sedentary (SED), (ii) treated with the antioxidant catechin (CAT), or (iii) voluntarily exercised (EX) from weaning (1-month old; mo) to 9 mo. Then, all mice aged sedentarily and were untreated until 12 mo.* Sod2* promoter methylation was similar in all groups in 9 mo but decreased (*p* < 0.05) in 12 mo SED mice only, which was associated with an increased (*p* < 0.05) transcriptional activity* in vitro*. At all ages, femoral artery endothelial function was maintained; this was due to an increased (*p* < 0.05) contribution of eNOS-derived NO in 12 mo SED mice only. CAT and EX prevented these changes in age-related endothelial function. Thus, a ROS-dependent epigenetic positive regulation of* Sod2* gene expression likely represents a defense mechanism prolonging eNOS function in aging mouse femoral arteries.

## 1. Introduction

In the vasculature, age-related failure to control reactive oxygen species (ROS) levels creates a state of oxidative stress that irreversibly damages endothelial cell functions and leads to senescence [1]. For this reason, various antioxidants have been shown to have a beneficial effect on endothelial function, particularly through activation of the ROS-sensitive nitric oxide pathway [2, 3]. In the vasculature, chronic exercise (EX) maintains an optimal cardiovascular profile, increases antioxidant defenses, and preserves vascular endothelial function [4–7]. The endogenous antioxidant enzyme manganese superoxide dismutase (MnSOD) maintains low levels of ROS; knockdown of MnSOD is deleterious and shortens lifespan, while its overexpression counteracts age-related cardiovascular damage [8, 9]. We previously observed that the expression of MnSOD was reduced in the aorta of 12 mo mice chronically treated with the antioxidant catechin (CAT) [10], but the mechanisms responsible for adaptive regulation to the redox environment are poorly understood. It is known that epigenetic DNA methylation regulates gene expression and is sensitive to the redox environment [5, 11, 12]. Studies have shown that methylation of various regions in the* SOD2* gene correlates with changes in MnSOD expression such as in ROS-rich cancer cells [13]. Thus, we hypothesized that ROS regulate MnSOD expression in vascular cells through an epigenetic mechanism, namely, DNA methylation. This would protect against oxidative stress and, ultimately, preserve endothelial function.

## 2. Materials and Methods

### 2.1. Animal Model

To test our hypothesis, C57Bl/6 mice (*n* = 10 in each group) were either left sedentary (SED), treated with CAT (30 mg/kg/d) to reduce ROS availability [10], or voluntarily exercised by access to a running wheel (EX) to preserve antioxidant defenses [14] from weaning to the age of 9 months (9 mo) [14]. Half of each group was then kept in sedentariness and untreated for an additional 3 months. Approval by the Montreal Heart Institute Animal Ethical Committee (#R2014-62-02) was given for all animal experiments. At 9 and 12 mo, the femoral feed artery was isolated after euthanasia under anesthesia for reactivity studies [15] and for DNA extraction and methylation quantification.

### 2.2. DNA Methylation Quantification

Bisulfite conversion of DNA was done using the EZ DNA Methylation-Gold kit (Zymo Research, Irvine, CA). DNA methylation was quantified by EpiTYPER assay (Sequenom, San Diego, CA), as previously described [16]. For this method, specific forward (aggaagagagATGTTAGGTTAGGTTTTAGGGAAGG) and reverse (cagtaatacgactcactatagggagaaggctTCCCCTATACCAAATTAATAAAAACC) primers were used to amplify the region of interest.

### 2.3. *In Vitro* Methylation

For* in vitro* methylation studies, constructions were done using the CpG-free and promoterless vector pCpG-free basic-Lucia (Invivogen, San Diego, CA) as the backbone. A 469-bp* Sod2* promoter region was amplified on mouse genomic DNA using forward BamHI (5′-ATTGGATCCTTTGCAGCTCACAGCCAGAGCTGGACA-3′) and reverse HindIII (5′-TAAAAGCTTAGCCAGCCACGCCCGCCGCCCCG-3′)-linked primers and subsequently inserted in the backbone using BamHI and HindIII restriction sites. Cloned vectors were isolated by Qiagen QIAprep Spin Miniprep kit (Qiagen). M.SssI CpG methyltransferase (New England Biolabs, Frankfurt, Germany) was used for* in vitro* methylation according to manufacturer's instructions. Methylated DNA was then purified using the QIAquick gel extraction kit (Qiagen) and quantified by NanoDrop (Thermo Scientific NanoDrop Products, Wilmington, DE). Methylation was confirmed by digestion with the methylation-sensitive restriction enzymes HhaI and HpaII. HEK293 cells grown to confluence on 96-well plates were transfected with the pCpG-free Sod2 vector using Lipofectamine 2000 (Invitrogen). Following 24 h transfection, luciferase activity was measured with the QUANTI-Luc reagent (Invivogen) by luminescence detection.

### 2.4. Statistical Analysis

Comparisons were done using 2-way ANOVA with Bonferroni posttests and the data were reported as means ± SEM.

## 3. Results

### 3.1. Catechin Treatment and Exercise Prevent Age-Induced* Sod2* Demethylation

**Figure 1:**
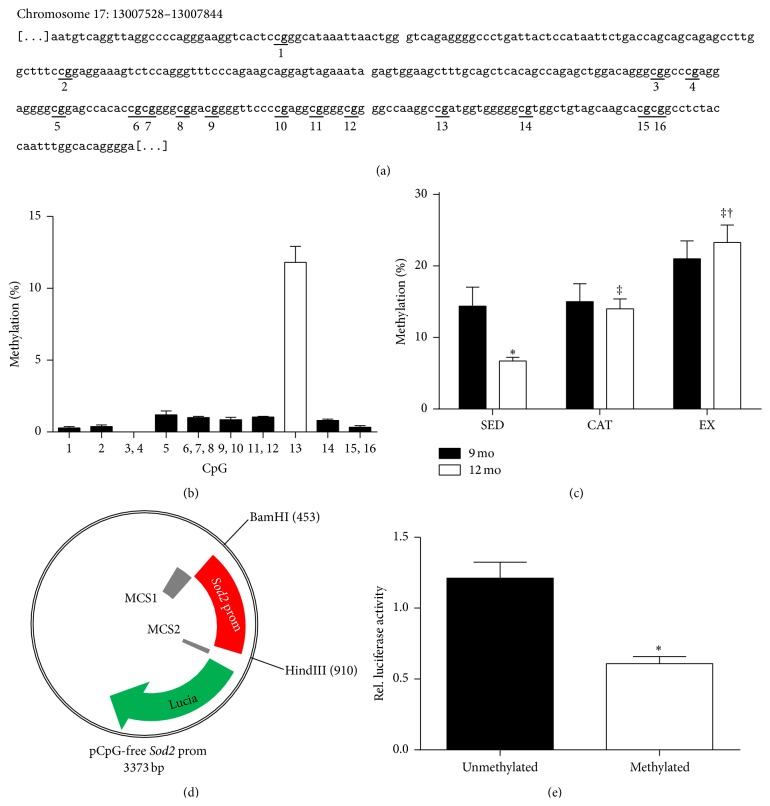
(a) Identification of all 16 CpG sites located in the targeted promoter region of the* Sod2* gene and (b) their methylation level independently of the experimental conditions tested. (c) Decrease with age (12 mo) in* Sod2* promoter methylation is prevented when mice had been treated by catechin, but more efficiently when voluntarily exercising. Methylation percentage of* Sod2* CpG in the femoral artery from sedentary (SED), voluntary exercised (EX), and catechin-treated (CAT) mice at 9 mo and 12 mo. ^*∗*^ *p* < 0.05 compared with 9 mo; ^‡^ *p* < 0.05 compared with SED; ^†^ *p* < 0.05 compared with CAT; *n* = 3–7 (two-way ANOVA, Bonferroni posttests). (d) Representation of the pCpG-free* Sod2* construct. (e)* In vitro* methylation decreases gene activity in a luciferase reporter assay. Luciferase activity was measured following transfection of HEK293 cells with the unmethylated or methylated pCpG-free Sod2 plasmid. ^*∗*^ *p* < 0.05, *n* = 3.

First, we studied the regulation of* Sod2* in segments of femoral arteries by targeting a DNA region in the promoter enriched in methylable CpG sites previously identified using the UCSC Genome Browser (http://genome.ucsc.edu/) (Figure 1(a)). Out of the 16 CpG sites found in this region, only CpG #13 showed detectable levels of methylation (>2%) (Figure 1(b)). The novel CpG site identified in our study complements the various CpG methylation sites previously characterized within the human* SOD2* gene (reviewed in [13]). Methylation of this site was similar between the 3 groups of 9 mo mice (Figure 1(c)). When compared to their respective 9 mo group, only 12 mo SED mice showed a 53% decrease in CpG #13 methylation, while CAT and EX mice remained unchanged with age. When comparing methylation levels at 12 mo, the EX group was the highest, the CAT group was intermediate, and the SED group was the lowest (*p* < 0.05; Figure 1(c)). Therefore, demethylation of this CpG seems sensitive to the increase of ROS occurring with age and it can be prevented by CAT and EX; clearly, EX initiated during early life is more efficient than CAT at maintaining high methylation level in the promoter, suggesting a better regulation of the redox environment in chronically exercising mice, as previously suggested [14].

### 3.2. *In Vitro* Methylation Decreases Expression

We then tested whether methylation of the promoter regulates* Sod2* gene expression. Methylation of the promoter of the CpG-free* Sod2* gene construct (Figure 1(d)) led to a significant decrease in luciferase transcriptional activity compared to the unmethylated promoter in HEK293 cells (Figure 1(e)), confirming the regulatory role of this CpG in* Sod2* transcription. It is known, indeed, that methylation of different sites in the* Sod2* promoter region inhibits transcription by blocking binding of transcription factors [17, 18].

### 3.3. Catechin Treatment and Exercise Prevent the Altered Vascular Function with Age

**Figure 2:**
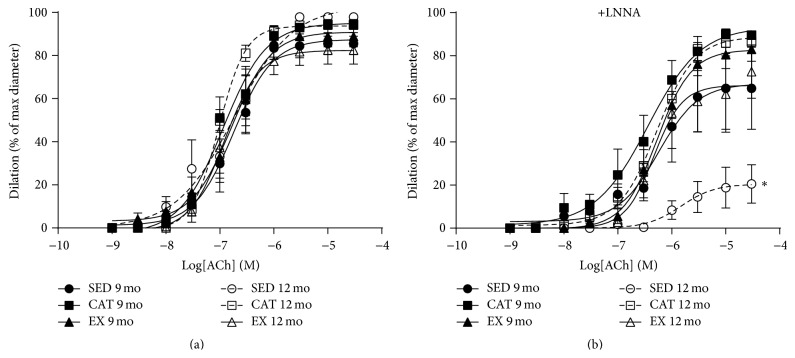
Catechin treatment and voluntary exercise prevent age-induced alterations in endothelial function. ACh-induced dilation of mouse femoral artery from sedentary (SED), voluntary exercising (EX), and catechin-treated (CAT) mice at 9 mo and 12 mo. Measurements were made in (a) basal conditions or (b) in the presence of N-nitro-L-arginine (LNNA; 100 *μ*M). ^*∗*^ *p* < 0.05, *n* = 3–9.

**Figure 3:**
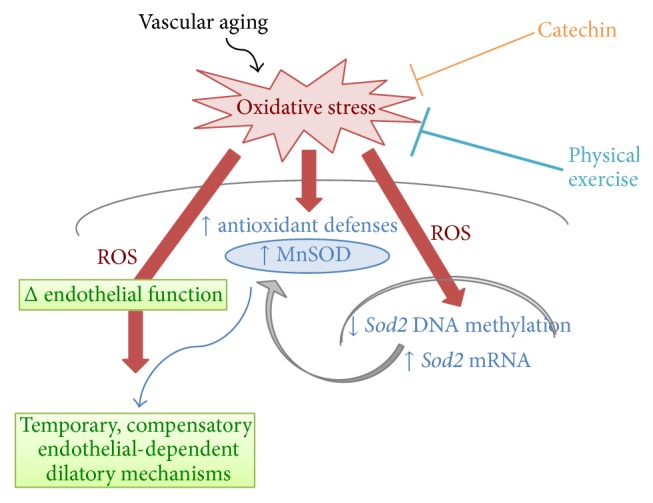
Schematic representation of redox-dependent* Sod2* promoter methylation in aging vascular cells. Aging is associated with a rise in oxidative stress, which favours increased MnSOD antioxidant defense, at least partially* via* a lower methylation of* Sod2*; a rise in MnSOD leads to a temporary, compensatory NO-dependent relaxing pathway that preserves endothelial function. This cascade is not observed in mice exposed to a protective cardiovascular environment provided by chronic treatment with the antioxidant catechin or long-term exposure to physical exercise.

Finally, we investigated the impact of* Sod2* methylation on the endothelial function of isolated and pressurized femoral artery segments. Endothelium-dependent dilations induced by increasing concentrations of acetylcholine were similar in arteries isolated from 9 and 12 mo mice (Figure 2(a)). After inhibition of NO production with LNNA, however, dilations tended to be lower at 9 mo and were significantly reduced at 12 mo in arteries isolated from SED mice only (Figure 2(b)). These results are indicative of the age-dependent loss of a highly ROS-sensitive dilatory factor that we previously identified in these arteries as a 17-octadecynoic acid (cytochrome P450/epoxygenase inhibitor) sensitive endothelium-derived hyperpolarizing factor (EDHF) [15]. We propose that NO production is increased to maintain the overall ability of the vessel to dilate in order to compensate for the loss of EDHF; because NO is susceptible to ROS inactivation, although to a lesser extent than EDHF in these arteries [15], an efficient antioxidant environment is required to preserve NO bioavailability [4]. These results, in parallel with the changes in methylation of* Sod2* promoter, suggest that the SED-associated accumulation of ROS in the femoral artery could stimulate the expression of MnSOD as a stress resistance mechanism in an attempt to maintain optimal endothelial function. In addition, they suggest that both CAT and to a greater extent regular EX delay the need for this adaptive epigenetic regulation to a later age by maintaining low oxidative stress. This hypothetical cascade of events is illustrated in Figure 3.

## 4. Discussion

In summary, our data confirm that vascular cells adapt to the changes in environment to limit stress by regulating the expression of defense mechanisms [19]. The current work extends from our two previous studies [14, 15] and suggests that ROS may trigger the transcriptional activity of the* Sod2* gene by epigenetic regulation of promoter activity through demethylation of at least one relevant CpG site.

Like others [20, 21], we identified an epigenetic mechanism responsible for* Sod2* expression and, to our knowledge, methylation at this specific site has never been reported. Because of the small size of the isolated vessels, it was not possible to extract DNA and proteins or DNA and mRNA from the samples. This is the main limitation of the study. In addition to DNA methylation, femoral arteries were also used, in the same mice, to assess endothelial function in isolated pressurized segments, further limiting the amount of tissue available. Because of this technical limitation, MnSOD expression was not quantified. Without the direct measurement of MnSOD expression in the vessel, the* in vitro* methylation assay was required to confirm the regulatory function of this methylation site (Figure 1(e)). The results obtained by this approach confirm that methylation of this site inhibits gene activity and allows us to speculate that in femoral arteries of aged mice any changes in the* Sod2* gene methylation can translate into changes in MnSOD expression. In addition, in a previous study using identical experimental groups of mice [14], we measured MnSOD activity in the aorta: at the age of 9 months, no difference in MnSOD activity was observed among control, CAT-treated, or exercised mice, data that are in agreement with similar* Sod2* methylation levels among groups in 9-month mice. At the age of 12 months, we previously observed that MnSOD activity tended to increase in the untreated sedentary group of mice, while MnSOD was the lowest in the exercising mice [14]; again, these results fit with a lower level of* Sod2* methylation in 12-month control sedentary mice and with a higher methylation level in 12-month exercised mice as shown in this study. However, despite the general agreement between aortic MnSOD activity and the methylation data obtained in femoral arteries, no direct correlation should be made.

It has been shown that vascular MnSOD expression is directly correlated with the preservation of vascular function [22, 23]. We considered the possibility that, while ROS trigger damage to the vascular endothelium, they also could signal for the upregulation of antioxidant defense mechanisms. We hypothesized that an epigenetic mechanism would be at the root of this regulatory adaptive process (Figure 3). Our results tend to support our hypothesis by showing that* Sod2* promoter methylation is regulated in response to the physiological context of aging vascular cells. Indeed, the observed decrease in* Sod2* methylation in 12-month control mice likely translates into higher MnSOD expression, in response to an age-dependent rise in oxidative stress. In contrast, the level of* Sod2* methylation rose in 12-month CAT-treated or exercised mice, suggesting lower MnSOD expression and activity in the antioxidant environment provided by both long-term catechin treatment and exercise. The reduction in promoter methylation of* Sod2* may therefore represent an adaptive protective effect in order to upregulate MnSOD expression.

In parallel with decreased* Sod2* methylation, aged SED mice suffer from an altered endothelial function, revealed by a NO-dependent dilatory pathway not observed in CAT-treated or exercised mice, suggesting that a shift in the redox environment requires the expression of MnSOD antioxidant enzyme to maintain the overall endothelial function, at least temporarily. Catechin treatment and chronic exercise were able to prevent the age-induced effects on the vasculature together with the changes in* Sod2* methylation. We therefore believe that, in accordance with our working hypothesis, aging is associated with a rise in oxidative stress, which favours increased MnSOD antioxidant defense, at least partially via a lower methylation of* Sod2*, and leads to a compensatory NO-dependent relaxing pathway. This cascade is not observed in mice exposed to a protective cardiovascular environment. Further studies are required to determine at what age (>12 months) MnSOD antioxidant defense fails and oxidative damage compromises the compensatory endothelial relaxing pathway.

We hypothesized that the molecular signal responsible for the changes in DNA methylation is free radicals that accumulate with age in the vasculature: high oxidative stress leads to low DNA methylation, while low oxidative stress leads to high DNA methylation of* Sod2*, permitting fine-tuning in antioxidant enzyme expression according to the redox environment (Figure 3). Accordingly, the presence of the antioxidant catechin prevented the changes in DNA methylation. Surprisingly, exercise is associated with even higher methylation levels when compared to catechin, suggesting that exercise further decreases free radicals production, which would require even less MnSOD expression. This difference between catechin and exercise is in accordance with our previous study showing that exercise was more efficient at protecting cerebrovascular endothelial function than catechin treatment [14]. Relevant to this observation, the beneficial effects of antioxidant supplementation on the treatment of ROS-elevated diseases remain unclear, as reported by large-scale clinical trials [24].

Further studies are required to confirm and understand the regulatory role of free radicals on DNA methylation, as these interpretations are speculative.

In C57Bl/6 mice, 9 months of age is the tipping point for the maintenance of the endothelial function, the maturation of the defense mechanisms responsible for endothelial stress resistance being largely dependent on the early life diet and exercise environment [10, 14]. Our study provides, therefore, new insights into the mechanisms responsible for stress resistance by demonstrating that ROS may stimulate the expression of an adaptive defense system by regulating* Sod2* gene expression through methylation of the promoter. Whether beneficial interventions influence* Sod2* methylation in a direct manner or rather through the inhibition of ROS-driven epigenetic changes is unknown. Further studies are required to understand the mechanisms by which ROS interact with antioxidant enzymes regulation, likely involving the dual DNA methyltransferase and histone-modifying enzymes [25].

## 5. Conclusions

In the femoral artery of aging mice, a decrease in* Sod2* methylation can be prevented by either polyphenol supplementation or chronic exercise. This suggests a novel mechanism by which the expression of this antioxidant enzyme is regulated in response to a change in the redox environment.

## Abbreviations

EDHF: Endothelium-derived hyperpolarizing factor
H_2_O_2_: Hydrogen peroxide
MnSOD: Manganese superoxide dismutase
NO: Nitric oxide
ROS: Reactive oxygen species.
